# Locally Deployed Large Language Models for AI-Assisted Outpatient Prescription Review: Crossover Study

**DOI:** 10.2196/97520

**Published:** 2026-09-14

**Authors:** Zhengyue Liu, Yi Ding, Jingxia Chen, Ziqiang Yan, Xuxi Cheng, Wei Zhou, Peng Fu, Zhuo Wang

**Affiliations:** 1 School of Clinical Pharmacy, Shenyang pharmaceutical university Shenyang China; 2 Department of Pharmacy, Shanghai Changhai Hospital, The First Affiliated Hospital of Navy Medical University Shanghai China

**Keywords:** large language model, prescription review, human–artificial intelligence collaboration, human-AI collaboration, on-premise deployment, knowledge augmentation, Ollama

## Abstract

**Background:**

Pharmacists’ prescription review is key to medication safety, but rising numbers of outpatient prescriptions and expanding formularies leave less time per case, increasing error risk. Large language models (LLMs) show promise, yet two barriers hinder routine use: first, most systems rely on cloud-based commercial models, risking data breaches by transmitting protected patient information externally, and second, retrieval-augmented generation (RAG) pipelines used to reduce hallucinations depend on complex text vectorization and vector databases, which hospitals with limited IT resources struggle to build and maintain.

**Objective:**

This study aimed to evaluate the feasibility and usefulness of a locally deployed, knowledge-augmented LLM as a decision-support tool for pharmacist-led outpatient prescription review.

**Methods:**

The open-source Qwen3-14B model was deployed on a hospital intranet server using the Ollama framework. A structured knowledge base derived from drug package inserts served as the principal reference and supported lightweight knowledge augmentation through exact-match injection. A 2-period crossover design was used: 2 pharmacists independently reviewed 213 outpatient prescriptions under both unaided and AI-assisted conditions, yielding paired unaided and collaborative results for each prescription. Plain AI review and knowledge-augmented AI review were also run on the same 213-prescription test set to quantify hallucination rates, and stand-alone AI performance was assessed against the reference standard. The reference standard was established by independent consensus between two supervising pharmacists, with disagreements adjudicated by a deputy chief pharmacist. Accuracy, sensitivity, and specificity were compared between conditions using paired McNemar tests, and review time was compared using the Wilcoxon signed-rank test.

**Results:**

Overall review accuracy was 97.2% (207/213, 95% CI 94.0%-98.7%) in the human-AI collaborative condition vs 82.6% (176/213, 95% CI 77.0%-87.1%) in the pharmacist-alone condition (*P*<.001). Sensitivity was 98% (61/62) vs 55% (34/62), and the false-negative rate fell from 45% to 2% (*P*<.001). Specificity did not differ significantly (146/151, 96.7% vs 142/151, 94%; *P*=.29). Knowledge augmentation reduced the model hallucination rate from 19.7% (42/213 prescriptions) to 4.7% (10/213), an absolute reduction of 15.0 percentage points (relative reduction 76.2%; *P*<.001). Collaborative review reduced the mean per-prescription review time from 2.33 (SD 0.97) to 1.12 (SD 0.49) minutes (approximately 51.9% reduction; Wilcoxon signed-rank *Z*=−12.65; *P*<.001; *r*=0.87).

**Conclusions:**

A locally deployed, knowledge-augmented LLM used as a pharmacist-supervised prescreening tool was associated with substantially higher accuracy and sensitivity in outpatient retrospective prescription review, while keeping all prescription data within the hospital network. Locally deployed open-source models may offer hospitals a privacy-preserving and practical route to AI-assisted pharmacy decision support.

## Introduction

Prescription review is fundamental to ensuring medication safety and rational drug use in health care institutions. China’s Hospital Prescription Review Management Standards (Trial) designates pharmacists as the primary agents of prescription review across all levels of health care facilities [1]. However, as outpatient prescription volumes and drug formularies steadily expand, pharmacists face growing pressure to evaluate indication appropriateness, dosing, drug interactions, and other factors within limited time, inevitably raising the risk of oversight [2]. Finding effective supportive tools to enhance review quality and efficiency while preserving the pharmacist’s central role has therefore become an urgent priority.

Since 2023, large language models (LLMs) have gained considerable traction in health care. Studies have demonstrated that LLMs can effectively encode clinical information and perform comparably to experts on medical question-answering tasks [3,4]. In the domain of medication safety, researchers have explored the capacity of LLMs to correct medication direction errors [5], identify drug-drug interactions [6], and review prescriptions [7]. Despite this progress, LLMs still fall short of matching pharmacist-level expertise. In a prospective crossover study, Ong et al [8] showed that combining LLM-based screening with physician review achieved significantly higher medication error detection rates than either method alone. LLMs thus appear better suited as a complement to clinical practice rather than a replacement.

Despite these advances, two critical gaps remain. First, nearly all existing studies rely on cloud-based platforms. Prescription data inherently contain legally protected patient information (eg, diagnoses and medication histories) that cannot be transmitted through external interfaces without substantial data breach and regulatory risk [9]. Second, the hallucination problem intrinsic to LLMs poses particular dangers in clinical settings [10]. Retrieval-augmented generation (RAG) is the prevailing strategy for mitigating hallucinations [11]; however, conventional RAG pipelines require text vectorization, embedding models, and vector databases, creating a significant technical barrier for health care facilities with limited informatics resources.

To address these gaps, we deployed the open-source Qwen3-14B model on a hospital intranet server via the Ollama framework and constructed a structured knowledge base from drug package inserts. Instead of the vectorization and semantic retrieval overhead of conventional RAG, we adopted exact-match injection for knowledge augmentation. Importantly, this study was not designed to evaluate stand-alone AI performance. We used a crossover design grounded in routine pharmacy workflows, comparing unaided pharmacist review with AI-assisted pharmacist review and systematically assessing the impact of knowledge augmentation on model hallucinations. This approach preserves regulatory compliance by maintaining pharmacists as the reviewers and reflects realistic implementation conditions. The aim of this study was to determine whether a locally deployed, knowledge-augmented LLM, serving as a pharmacist-supervised prescreening tool, improves accuracy, sensitivity, and efficiency of outpatient prescription review compared with unaided review, and to quantify the degree to which exact-match knowledge augmentation reduces hallucinations. We hypothesized that AI-assisted review would improve accuracy and sensitivity without meaningful loss of specificity, and that knowledge augmentation would substantially lower the hallucination rate relative to unaugmented AI review.

## Methods

### System Architecture and Knowledge Augmentation

The system adopts a browser or server architecture with a PHP (version 8.2.27; PHP Group) back end communicating with a locally deployed Ollama (version 0.10.1; Ollama Inc) instance serving Qwen3-14B (Alibaba Group; Q4_K_M quantization, greedy decoding, up to 5120 tokens per prescription) on the hospital intranet. All processing remained fully offline. The end-to-end pipeline and its comparison with conventional RAG are illustrated in Figures 1 and 2. Implementation details, including hardware specifications, are provided in Multimedia Appendix 1.

**Figure 1:**
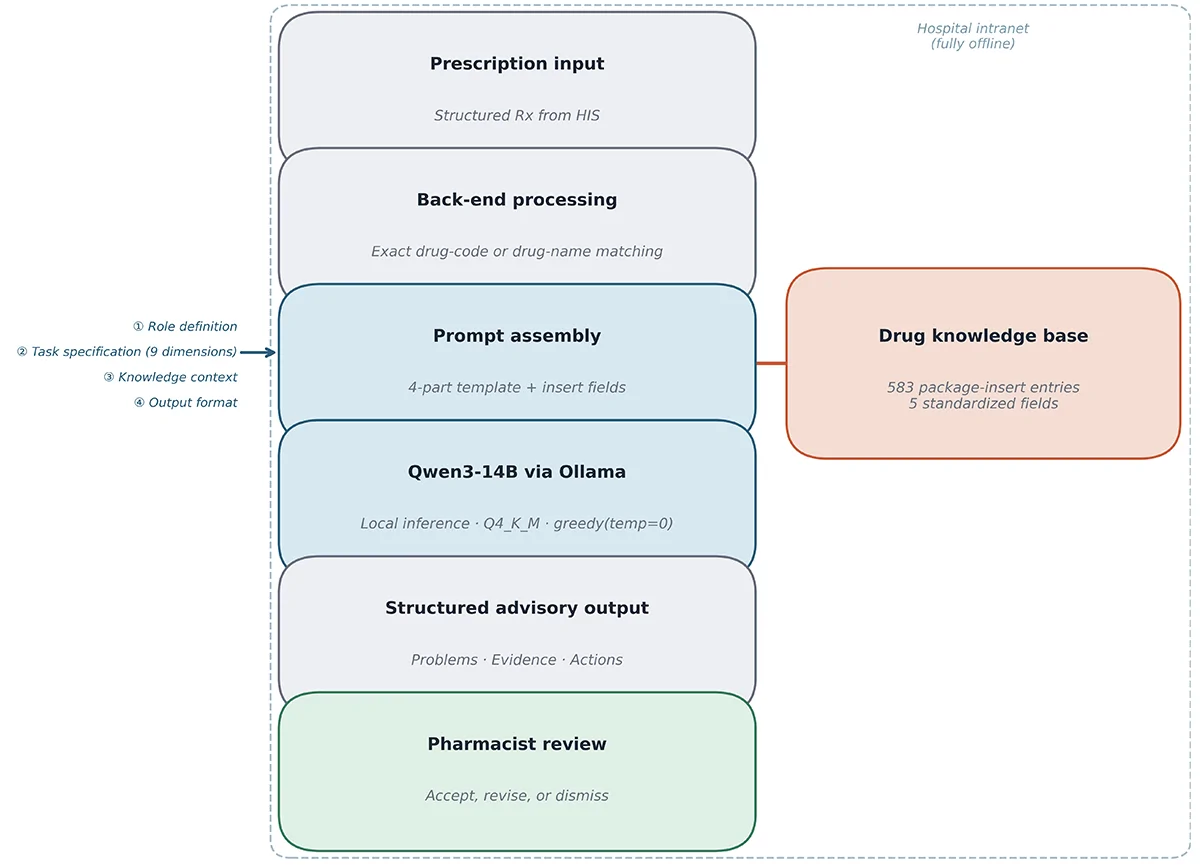
End-to-end knowledge-augmented review pipeline. The offline drug knowledge base feeds matched insert fields into prompt assembly; the local Qwen3-14B model performs inference and returns a structured advisory output. HIS: hospital information system.

**Figure 2:**
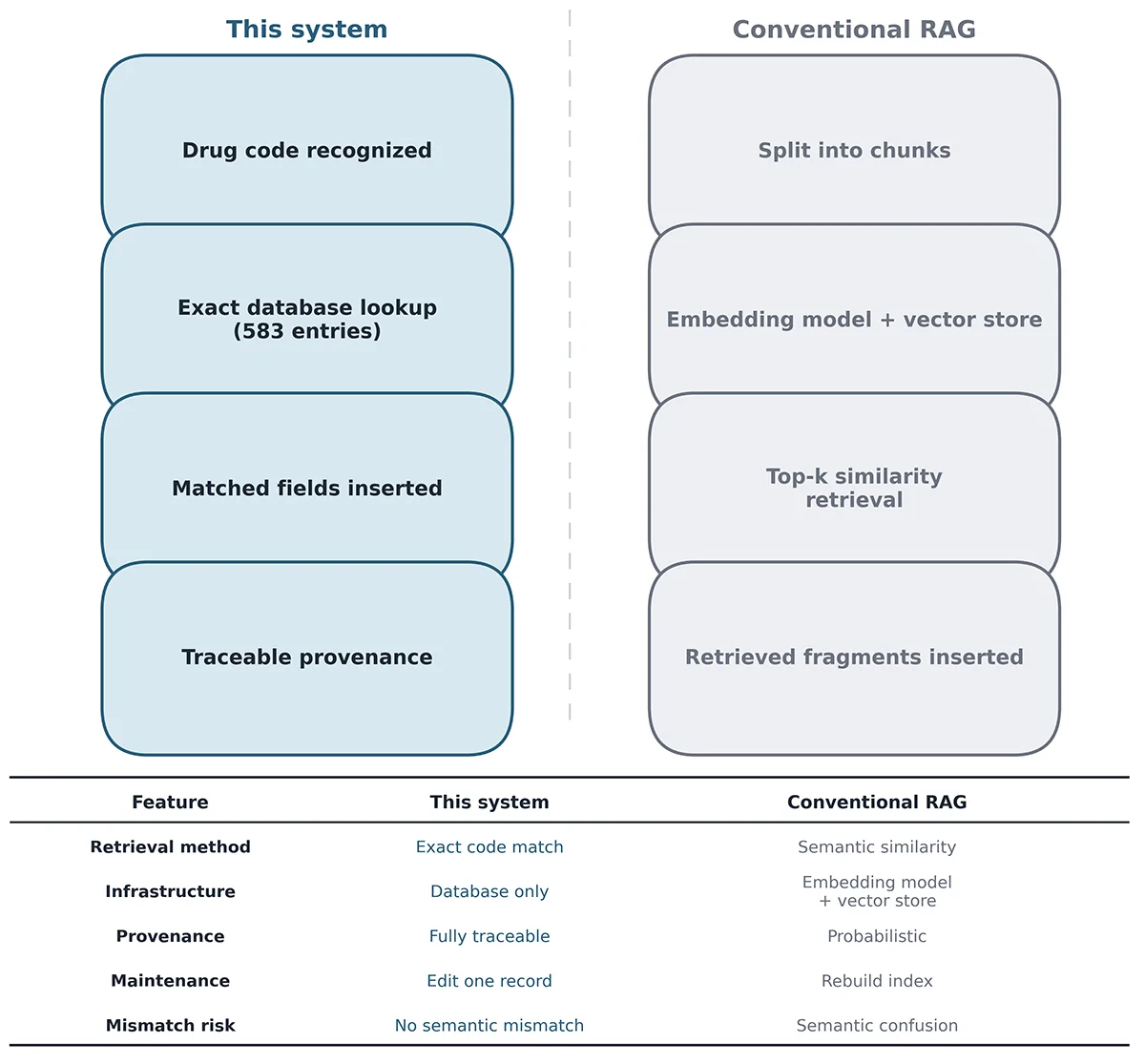
Deterministic exact matching vs conventional retrieval-augmented generation (RAG). Exact matching is traceable, needs no retraining, and removes the semantic mismatch risk of vector retrieval, although it still depends on accurate drug code correspondence; conventional RAG adds infrastructure and carries a risk of semantic mismatch.

Drug knowledge was sourced exclusively from package inserts, which serve as the legally binding reference under Chinese regulations, decomposed into 5 standardized fields (indications, dosage and administration, contraindications, interactions, and special-population precautions) across 583 entries. Upon recognizing a drug code, the system performed deterministic exact matching and injected the corresponding fields into the prompt. This approach avoids the need for an embedding model, vector store, and semantic mismatch risks inherent to conventional RAG; provenance is fully traceable, and updates require only editing a single database record. Because matching is keyed on the drug code rather than on free text, it removes the semantic retrieval errors of vector search, although it still depends on consistent coding; a brand-vs-generic naming difference or a coding inconsistency in the hospital information system could therefore prevent a match. Such cases are handled by the same safeguard applied to unmatched drugs: they are treated as uninjected and flagged for manual review, so a failed match surfaces as an explicit no-injection alert rather than a silent or erroneous injection. Drugs absent from the knowledge base were flagged for manual pharmacist review; all 213 test prescriptions were fully covered. We chose package inserts over subscription compendia such as Micromedex (Merative LP) or UpToDate. Although these compendia offer richer indication and dosage information, they require licensing and external connectivity, which is incompatible with the fully offline, privacy-preserving design at the core of our study.

The prompt comprised 4 components: role definition (advisory tool, not a decision-maker), task specification (9 review dimensions, including indication-diagnosis correlation, dosage, interactions, and special populations), injected package-insert content and review rules, and a structured output format (problem description, supporting evidence, and recommended action). The complete template is provided in Multimedia Appendix 2.

### Study Design

#### Population and Allocation

The population of the study was outpatient prescriptions from a tertiary teaching hospital in October and November 2025. Inclusion criteria were prescriptions with a minimum of 1 chemical or biological pharmaceutical drug. Exclusion criteria were traditional Chinese herbal decoction prescriptions. Following the screening process, 213 prescriptions were randomly sampled at a rate of 0.1%, as specified by the Hospital Prescription Review Management Standards [1].

The sample size was fixed by this administrative sampling rule, and no a priori power calculation was performed before the study. We did not conduct a post hoc power analysis, because observed power is a deterministic function of the observed *P* value and adds little information beyond the CIs. Instead, the precision of every estimate is conveyed by its Wilson-score 95% CI, and the fixed sample size is acknowledged as a limitation. As a planning reference only, the sample size formula for the McNemar test described by Lachin [12] indicates that, for a 2-sided α of .05, 80% power, and a between-condition accuracy difference of at least 10 percentage points, about 124 discordant-informative pairs would be required; the 213 paired observations available therefore provided a reasonable basis for the primary comparison. The therapeutic duplication (n=5, 2.3%) and dosage inappropriateness (n=11, 5.2%) subgroups contained too few cases for adequately powered testing and are reported as exploratory.

#### Crossover Design and Collaborative Workflow

A 2-period crossover design was used (Figure 3). The 213 prescriptions were randomly allocated to batch A (n=107, 50.2%) and batch B (n=106, 49.8%). Two junior pharmacists (P1 and P2; 1-2 years of experience) each performed 1 period of unaided review and 1 period of human-AI collaborative review, counterbalanced across batches with a 2-week washout interval, yielding 213 paired observations (1 unaided and 1 collaborative result per prescription).

**Figure 3:**
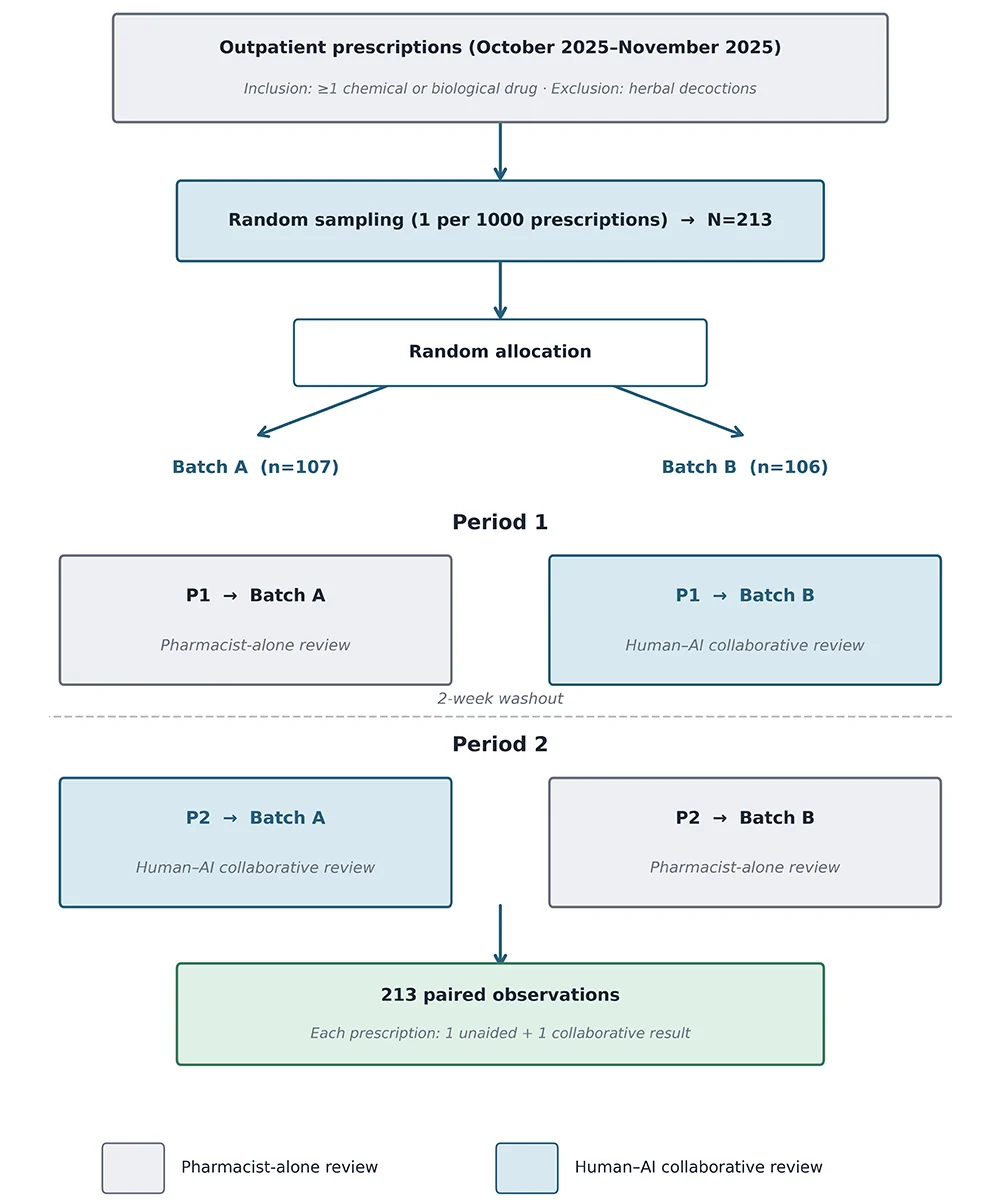
Two-period crossover design. Prescriptions were randomly split into 2 batches and counterbalanced across pharmacists and periods, separated by a 2-week washout.

In the collaborative condition (Figure 4), the pharmacist reviewed system-generated alerts, each linked to package-insert evidence, and exercised sole decision-making authority to accept, revise, or dismiss each flag or to add findings not identified by the system. Every alert disposition was recorded as accepted or overridden according to the reviewing pharmacist’s judgment. Overridden alerts were subsequently verified against the reference standard and classified as correct or incorrect. The system automatically recorded the review time of each prescription as the wall-clock interval from opening to submission, capturing end-to-end time at the prescription level rather than the drug-item level. Model inference for every prescription in a batch was completed beforehand as an offline preprocessing step, so that pharmacists reviewed previously generated outputs; the recorded review time therefore reflects the pharmacist’s interaction with the system and excludes model inference latency. Per-prescription review time served as the primary efficiency outcome.

**Figure 4:**
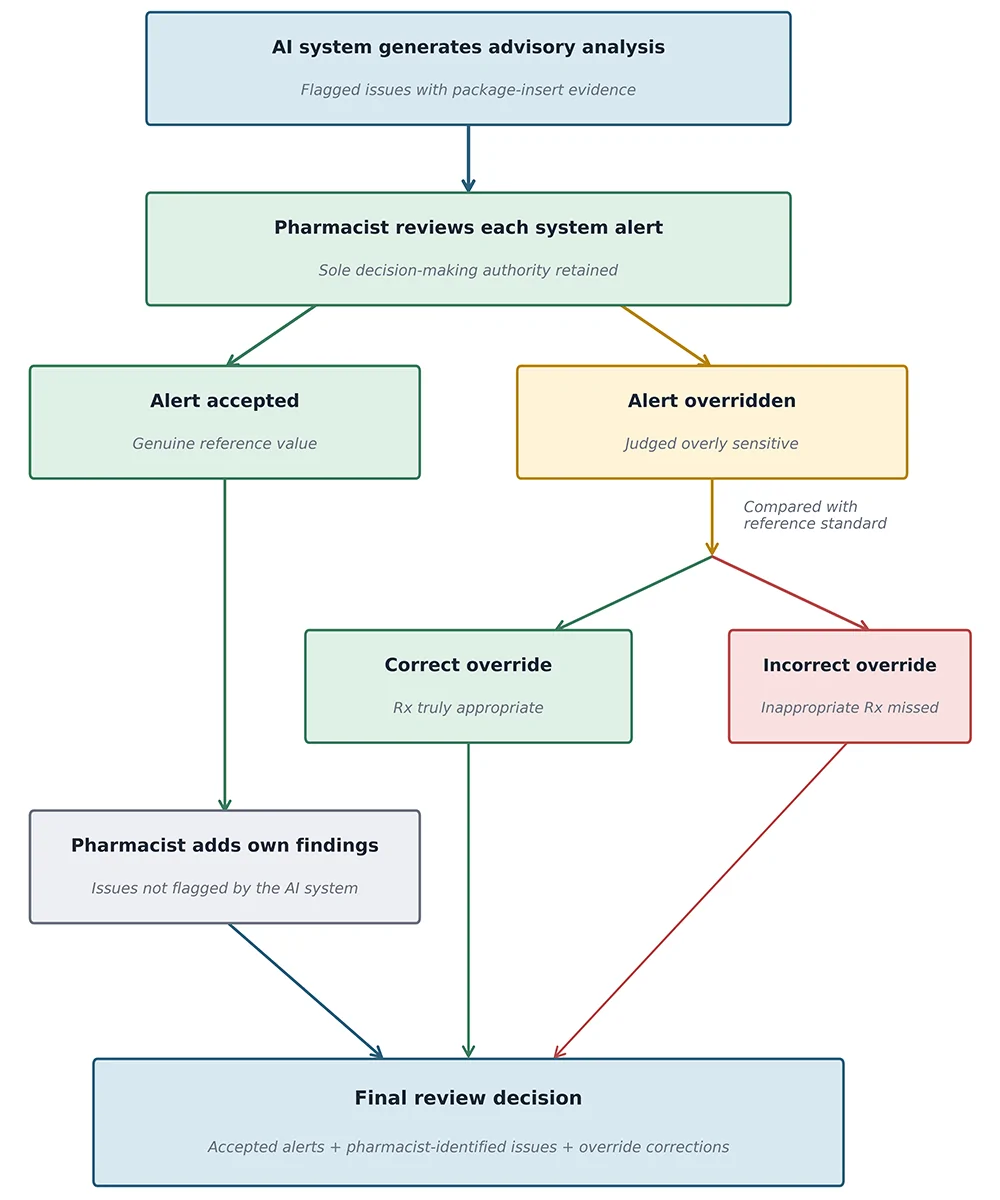
Human-AI collaborative workflow. The pharmacist reviewed each system alert, accepting or overriding it, and added any issues not flagged by the system; overridden alerts were compared with the reference standard.

#### Comparison of AI Review Modes

All 213 prescriptions were additionally processed in 2 stand-alone AI modes, plain (parametric knowledge only) and knowledge-augmented, to quantify the effect of knowledge injection on hallucinations. Hallucination was defined as any assertion contradicting the package insert or established clinical guidance, adjudicated at the prescription level by 2 senior pharmacists (disagreements resolved by a deputy chief pharmacist); Cohen κ quantified interrater reliability. The knowledge-augmented AI’s stand-alone diagnostic performance was also evaluated against the reference standard.

#### Reference Standard

Two supervising pharmacists (>5 years of prescription-review experience), blinded to all experimental outputs, independently assessed all 213 prescriptions; discrepancies were resolved by a deputy chief pharmacist. A sensitivity analysis included all prescriptions flagged by any condition but classified as appropriate by the expert panel for post hoc review (with blinding lifted); verified inappropriate prescriptions were added to an extended reference standard, and all analyses were repeated.

### Statistical Analysis

Baseline characteristics of the 213 prescriptions were summarized overall and by crossover sequence (P1 and P2), and between-sequence balance was tested with the Mann-Whitney *U* test for age and drug items per prescription (both nonnormal by the Shapiro-Wilk test) and the Pearson chi-square test for sex and age category.

The primary outcome was overall accuracy (correct classifications divided by total prescriptions); secondary outcomes included sensitivity, specificity, false-positive rate, false-negative rate, and mean review time. Paired conditions were compared with the McNemar test (Yates continuity correction; exact binomial McNemar test for small subgroups). A Mann-Whitney *U* test (with a *P*>.10 threshold) verified batch exchangeability before pooling periods. All proportions are reported with Wilson 95% CIs. No multiplicity correction was applied to exploratory end points. Analyses were performed in Python (version 3.10.2; Python Software Foundation). Additional statistical analysis details are provided in Multimedia Appendix 1.

### Ethical Considerations

This retrospective, noninterventional study analyzed existing, deidentified outpatient prescription records, with no contact with patients and no influence on clinical care. It therefore qualified for exemption from ethics committee review and individual informed consent under Article 32 of China’s 2023 Measures for Ethical Review of Life Science and Medical Research Involving Humans, and the institutional research-governance office confirmed this exemption (no case number applies, as no review was sought) [13]. Direct identifiers were removed during extraction, and only the fields required for analysis (patient age, sex, diagnosis, drug, dose, route, and frequency) were retained, yielding a dataset from which no individual can be identified. All processing occurred within the hospital intranet on access-controlled servers. No participants were recruited or compensated, and no image or supplementary file contains patient-identifiable information.

## Results

### Baseline Prescription Characteristics

The 213 prescriptions came from patients aged 1 to 95 years (median 57, IQR 38-69); 110 (51.6%) were female, and the median number of drug items per prescription was 2 (IQR 1-3). Sequences P1 (n=107, 50.2%) and P2 (n=106, 49.8%) were well balanced with respect to age (*P*=.86), age category (*P*=.33), sex (*P*=.73), and drug items per prescription (*P*=.33), supporting pooling across periods (Table 1).

**Table 1:** Demographic and clinical characteristics of the 213 outpatient prescriptions sampled at a tertiary teaching hospital in China (October 2025-November 2025) for a 2-period crossover study comparing unaided pharmacist review with human-AI collaborative review, overall and by crossover sequence.

Characteristics			Overall (N=213)		Sequence P1 (n=107)		Sequence P2 (n=106)		*P* value	
**Age (years)**										.86^a^
	Mean (SD)	53.5 (19.9)		53.0 (21.3)		54.1 (18.5)				
	Median (IQR)	57 (38-69)		57 (36-70)		56 (40-68)				
	Range	1-95		1-93		13-95				
**Age group (years), n (%)**										.33^b^
	Children (<18)	12 (5.6)		8 (7.5)		4 (3.8)				
	Adults (18-59)	106 (49.8)		49 (45.8)		57 (53.8)				
	Older adults (≥60)	95 (44.6)		50 (46.7)		45 (42.4)				
**Sex, n (%)**										.73^b^
	Male	103 (48.4)		50 (46.7)		53 (50)				
	Female	110 (51.6)		57 (53.3)		53 (50)				
**Drug items per prescription**										.33^a^
	Mean (SD)	2.16 (1.04)		2.07 (0.96)		2.25 (1.11)				
	Median (IQR)	2 (1-3)		2 (1-3)		2 (1-3)				

^a^Mann-Whitney *U* test; age and drug items per prescription were nonnormally distributed (Shapiro-Wilk *P*<.05 in ≥1 group).

^b^Pearson chi-square test.

### Comparison of Review Performance Between Groups

Before pooling the two periods, we examined whether the two prescription batches were comparable across sequences; given the prescription-level paired design, this is a check of batch and sequence comparability rather than a formal person-level carryover test. The Mann-Whitney *U* test, using the per-prescription sum of the two-period outcomes as the test statistic, showed no significant difference between sequences (batch A in period 1: n=107, 50.2%, vs batch B in period 2: n=106, 49.8%; *P*=.37). Because the paired unit was the prescription and the sequences corresponded to the 2 prescription batches, this result indicates that the batches were exchangeable with no detectable sequence-level effect; the 2 periods were therefore pooled for the main analysis.

Independent expert review and adjudication classified 62 (29.1%) of 213 prescriptions as inappropriate: 51 (24.0%) with an inappropriate indication, 11 (5.2%) with an inappropriate dosage, and 5 (2.3%) with therapeutic duplication (67 distinct errors in total, as some prescriptions had >1 problem). No prescription in the test set contained a clinically significant drug-drug interaction, a physicochemical incompatibility, or a confirmed contraindication or special-population error. Consequently, the system performance reported here pertains to indication, dosage, and duplication errors; its proficiency in detecting interactions, incompatibilities, contraindications, and special-population problems could not be validated in this sample, even though each of these dimensions is specified in the prompt (addressed in the Limitations). All subsequent findings are based on this reference standard, with between-condition performance summarized in Table 2.

**Table 2:** Prescription-review performance of unaided pharmacist review vs human-AI collaborative review on 213 outpatient prescriptions sampled at a tertiary teaching hospital in China (October 2025-November 2025) in a 2-period crossover study, using an independent expert reference standard (62 inappropriate prescriptions)^a^.

Groups	Accuracy (%)	Sensitivity (%)	Specificity (%)	False-positive rate (%)	False-negative rate (%)
Pharmacist alone	82.6	55	94	6	45
Human-AI collaborative	97.2	98	96.7	3.3	2
Chi-square test (*df*)	25.71 (1)	25.04 (1)	—^b^	—	25.04 (1)
*P* value	<.001	<.001	.29	.29	<.001

^a^Comparisons used paired McNemar tests with Yates continuity correction (213 paired observations). For accuracy, there were 33 discordant pairs that were correct only with collaboration and 2 that were correct only when unaided; for sensitivity and the false-negative rate, there were 27 and 0 discordant pairs, respectively. Accuracy denominators were 213; sensitivity, specificity, and the error rates used 62 inappropriate and 151 appropriate prescriptions, respectively.

^b^Specificity and false-positive-rate differences were not significant, and their test statistics are not reported.

Human-AI collaborative review significantly outperformed pharmacist-alone review in both overall accuracy and sensitivity (Table 2). Specificity did not differ between the 2 groups, indicating that AI support did not increase the misclassification of appropriate prescriptions as inappropriate and therefore added no meaningful false-positive risk.

To characterize individual pharmacist differences, Cohen κ was calculated for each pharmacist’s unaided review against the reference standard. Because P1 and P2 reviewed different prescription batches, the 2 pharmacists were not directly compared. These values therefore reflect criterion agreement with the reference standard. They do not represent interrater reliability between P1 and P2. P1 yielded κ=0.554 (95% CI 0.360-0.749; observed agreement 84.1%) and P2 yielded κ=0.520 (95% CI 0.284-0.751; observed agreement 81.1%). By the Landis and Koch classification, both fall within the moderate-agreement interval (κ range: 0.41-0.60). The two estimates were close (|Δκ|=0.034), indicating similar criterion agreement, although κ values obtained on nonoverlapping batches cannot establish agreement between the raters themselves. A related caveat concerns the paired analysis: because different reviewers performed the two conditions for any given prescription, the prescription-level differences carry a rater component, which the counterbalanced crossover design balances across conditions at the aggregate level.

Evaluated as a stand-alone classifier against the same reference standard, the knowledge-augmented AI flagged 77 prescriptions and achieved a sensitivity of 100% (62/62; 95% CI 94.2%-100%), a specificity of 90.1% (136/151; 95% CI 84.3%-93.9%), and an accuracy of 93% (198/213; 95% CI 88.7%-95.7%). The AI alone was thus highly sensitive but less specific than the collaborative condition, and its stand-alone accuracy (198/213, 93%) was lower than that achieved by the collaborative workflow (207/213, 97.2%). By retaining almost all the AI’s sensitivity while the pharmacist’s judgment raised specificity, the collaborative condition attained the highest overall accuracy, illustrating the complementary mechanism underlying the collaborative benefit. The increase in specificity, from 90.1% (136/151) in the stand-alone system to 96.7% (146/151) in the collaborative condition, was driven by pharmacist filtering of the model’s false alarms: of the 15 (7%) appropriate prescriptions that the AI overflagged, the pharmacists correctly dismissed 10 (4.7%) and accepted 5 (2.3%), while overriding 1 genuine alert (the single false negative in the collaborative arm).

### Detection of Different Problem Types

Subgroup analysis showed that the magnitude of AI-assisted improvement varied by problem type (Table 3). Where sample sizes were adequate, the collaborative group consistently outperformed the pharmacist-alone group. Identification of inappropriate indications rose from 61% (31/51) to 98% (50/51; *P*<.001), and identification of inappropriate dosages rose from 46% (5/11) to 100% (11/11; *P*=.03). Both categories rely on relatively objective criteria that can be verified against package inserts or diagnostic information, so the system’s knowledge base effectively compensated for gaps in pharmacist recall, yielding the clearest benefit from collaboration. Detection of therapeutic duplication improved from 20% (1/5) to 100% (5/5), but the small sample (N=5) precluded statistical significance (exact McNemar *P*=.13); this result is reported for reference only.

**Table 3:** Detection rates for each type of prescription problem under unaided pharmacist review and human-AI collaborative review among 213 outpatient prescriptions from a tertiary teaching hospital in China^a^.

Problem types	Pharmacist-alone, n (%)	Collaborative, n (%)	McNemar *P* values
Indication inappropriateness (n=51)	31 (61)	50 (98)	<.001
Dosage inappropriateness (n=11)	5 (46)	11 (100)	.03
Therapeutic duplication (n=5)	1 (20)	5 (100)	.13

^a^All subgroup sample sizes were small (n=5-51); exact McNemar tests (binomial exact) were used for paired comparisons. Detection rates were calculated per error: the denominators (n=51, 11, and 5) sum to the 67 distinct errors contained in the 62 inappropriate prescriptions, whereas the performance metrics in Table 2 were calculated per prescription. The collaborative detection counts (50+11+5=66) therefore differ from the 61 true-positive prescriptions implied by Table 2 because some prescriptions carried >1 error.

### Effect of Knowledge Augmentation on Model Hallucinations

On the same 213-prescription test set, plain AI review produced 101 marked events, of which 42 were adjudicated as hallucinations affecting 42 distinct prescriptions, a per-prescription hallucination rate of 19.7% (42/213). All 42 hallucinations fell into 3 categories: erroneous dosing advice (45%, 19/42), fabricated drug interactions absent from the package insert (38%, 16/42), and false indications (17%, 7/42). After knowledge augmentation, the system produced 94 marked events with only 10 adjudicated hallucinations across 10 prescriptions (4.7%, 10/213), an absolute reduction of 15.0 percentage points and a relative reduction of 76.2%. Because both modes processed the same 213 prescriptions, the comparison was paired: hallucinations occurred only in plain mode for 33 (15.5%) prescriptions and only in augmented mode for 1 (0.5%), while 9 (4.2%) prescriptions had hallucinations in both modes and 170 (79.8%) had hallucinations in neither mode. The resulting McNemar *χ*^2^_1_ was 28.26 (*P*<.001). Among the 10 residual hallucinations in augmented mode, 8 (80%) were drug interaction errors, mostly in complex prescriptions with >2 coprescribed drugs, suggesting the model may still extrapolate beyond injected knowledge when package-insert information for several drugs overlaps.

Interrater reliability of hallucination adjudication was good, with Cohen κ of 0.858 (95% CI 0.756-0.959) for plain mode and 0.810 (95% CI 0.627-0.992) for knowledge-augmented mode, indicating substantial to almost perfect agreement.

### Review Efficiency

The pharmacist-alone condition required 496.3 minutes to complete the full batch, whereas the human-AI collaborative condition required 238.6 minutes (N=213 prescriptions), yielding per-prescription review times of 2.33 (SD 0.97; median 1.95, IQR 1.47-2.92) minutes and 1.12 (SD 0.49; median 0.92, IQR 0.70-1.45) minutes, respectively. The mean within-prescription reduction was 1.21 (SD 0.50, 95% CI 1.14-1.28) minutes, representing an approximately 51.9% decrease under the collaborative condition, with shorter times observed in all 213 paired prescriptions. As the paired differences deviated from normality (Shapiro-Wilk *W*=0.91; *P*<.001), the primary comparison used the Wilcoxon signed-rank test, which confirmed significantly shorter review times in the collaborative condition (*Z*=−12.65; *P*<.001; *r*=.87); a paired *t* test produced a consistent result (t_212_=35.34; *P*<.001; Cohen *d_z_*=2.42, a very large effect).

Two factors likely contributed: the system preselected potentially problematic prescriptions, enabling pharmacists to focus on higher-risk cases, and relevant package-insert information was retrieved automatically, eliminating manual reference lookup. Timing was recorded at the prescription level rather than the individual drug-item level, so the estimates reflect end-to-end review time per prescription.

### Pharmacist Acceptance and Feedback on System Suggestions

When working collaboratively, the system sent a warning of a possible problem with 77 prescriptions. The reviewing pharmacist accepted 86% (66/77) of these alerts and overrode the remaining 14% (11/77) as overly sensitive false alarms. Acceptance records the pharmacist’s disposition rather than verified clinical validity. As noted previously, 5 (7.6%) of the 66 accepted alerts concerned prescriptions that the reference standard ultimately classified as appropriate. Of the 11 overrides, 10 (90.9%) proved correct on verification against that standard; the single incorrect override produced the only false negative in the collaborative group. During unstructured verbal debriefings after the final review session, both participating pharmacists expressed willingness to use the system in routine practice, remarking that it eased the burden of manual reference lookup and the stress of possible omissions.

### Sensitivity Analysis

Some prescriptions flagged by experimental arms had been classified as appropriate under the reference standard. To assess whether such omissions affected the conclusions, these cases were returned to the expert panel for post hoc reevaluation. Three were confirmed as inappropriate and added to an extended reference standard (65 inappropriate prescriptions in total). Because both the pharmacist-alone and human-AI teams had correctly identified all 3, the discordant-pair structure between groups was unchanged.

Under the expanded standard, the pharmacist-alone group achieved 84% accuracy (95% CI 78.5-88.3) and 57% sensitivity (95% CI 44.8-68.2), while the collaborative group reached 98.6% (95% CI 95.9-99.5) and 98% (95% CI 91.8-99.7), respectively. The McNemar test statistic remained identical to the main analysis (χ^2^=25.71; *P*<.001), confirming that the overall conclusions are robust to minor imperfections in the reference standard.

## Discussion

### Principal Findings

In this 2-period crossover study, pairing a locally deployed, knowledge-augmented LLM with pharmacist review substantially improved outpatient prescription review. Overall accuracy rose from 82.6% with unaided review to 97.2% with collaborative review, and sensitivity rose from 55% to 98%, while specificity was essentially unchanged; the gain in error detection was therefore not obtained at the cost of additional false alarms. Exact-match knowledge augmentation lowered the model hallucination rate from 19.7% to 4.7%. Collaborative review was also markedly faster, with mean per-prescription review time decreasing from 2.33 (SD 0.97) minutes to 1.12 (SD 0.49) minutes (≈51.9% reduction). Taken together, these findings indicate that a modest, locally hosted open-source model, used as a pharmacist-supervised prescreening tool, can meaningfully strengthen medication safety review without transmitting prescription data outside the hospital network.

### Rationale and Significance of Local-Deployment Human-AI Collaboration

Using a crossover design, we compared unaided pharmacist review with review assisted by a locally deployed LLM. The collaborative mode achieved substantially higher accuracy, sensitivity, and efficiency, with the pharmacist retaining decisional authority and the LLM serving as a prescreening agent. This copilot approach is consistent with Ong et al [8], who found that collaborative review outperformed either the model or the clinician alone, although their work relied on cloud-based GPT-4. Our study extends this rationale by relocating deployment from the cloud to the hospital intranet. Because Ollama keeps prescription data within the institutional network, it addresses the data-privacy requirements of Chinese medical facilities [9]. These findings suggest that effective human-AI cooperation in pharmacy need not depend on commercial cloud models, since locally deployed open-source solutions can deliver comparable collaborative benefits while preserving privacy.

Prior literature on LLMs in pharmacy has focused largely on isolated model evaluation, including drug interaction identification [6], benchmarking against clinical pharmacists [7], community pharmacy decision support [14], and real-world drug information accuracy [15]. These studies collectively show that ChatGPT responses are often incomplete or contain errors. Although they delineate the performance boundaries of LLMs, none addresses the more practically meaningful question of whether pharmacist work is improved by LLM assistance. By taking the pharmacist-LLM dyad as the unit of evaluation, our study quantifies net real-world benefit and thereby complements the existing evidence base.

### Technical Characteristics of the Knowledge Augmentation Strategy and Its Hallucination-Mitigation Mechanism

Conventional RAG is significantly different from the exact-match injection approach. The clinical decision-support system outlined by Ong et al [8] involves document chunking, vector indexing, and semantic retrieval, a pipeline that incurs substantial development and maintenance costs. We use the unique nature of drug identifiers to minimize knowledge retrieval to an individual deterministic database search and avoid the need for embedding models and vector storage. In prescription review in particular, the query goal is a clear-cut drug name as opposed to free text; exact matching is thus more reliable than fuzzy semantic retrieval and far less prone to noise.

The assessment of hallucination provides additional support regarding the mechanism of the collaboration effect. Knowledge augmentation lowered the hallucination rate from 19.7% to 4.7%, corresponding to a relative reduction of approximately 76.2%, which indicates that exact-match injection meaningfully narrows the model’s output space and confers adequate trustworthiness for system-generated suggestions for routine pharmacist consultation. This further supports the finding that the acceptance rate in the collaborative group was 86%: a small hallucination rate is required so that pharmacists could trust and use system outputs effectively. It should also be noted that the evaluation of hallucinations was intended as an explanatory analysis in the current research; that is, it aims to elucidate the technical basis of the benefit of collaboration but not to act as an isolated main end point equivalent to accuracy and sensitivity.

### Clinical Effectiveness and Translational Value of the Collaborative Mode

The collaborative mode demonstrated its principal value in sensitivity. The miss rate for inappropriate prescriptions fell from 45% under pharmacist-only review to 2% with AI support. System prescreening reminders served as a safety net for lapses arising from heavy workloads or imperfect recall of drug-specific information during independent review. Specificity remained high in both groups (142/151, 94% vs 146/151, 96.7%) with no statistically significant difference, indicating that AI support added no meaningful false-positive risk. The pharmacists’ appraisal of system alerts was nonetheless imperfect: two-thirds of the model’s false alarms were correctly dismissed (10/15, 66.7%), while the remaining third (5/15, 33.3%) were accepted. This finding aligns with Ong et al [8], who reported that collaborative review does not increase false positives, and with Singhal et al [3], who showed that LLMs can perform at an expert level when provided with adequate contextual knowledge. Subgroup analysis revealed the greatest gains in knowledge-intensive, rule-based domains such as indication and dosage appropriateness, whereas improvements were smaller for judgments requiring integration of individualized patient information, defining both the current scope of the system and directions for future development.

From a clinical standpoint, raising sensitivity from 55% to 98% carries substantial weight. As a post hoc quality assurance measure, prescription review derives its core value from systematic identification of irrational drug use and the continuous feedback that subsequently shapes prescribing behavior. A high miss rate directly undermines this loop: undetected inappropriate prescriptions never enter quality control, the prescribing physicians receive no corrective feedback, and the same errors tend to recur. Reducing the false-negative rate from 45% to 2% produces a more representative profile of prescribing problems in review outputs, offering a more accurate basis for departmental feedback and behavioral intervention. Over time, improved detection should reinforce a positive review-feedback-improvement cycle and curtail inappropriate prescribing at its source. The benefit is particularly relevant for primary care institutions, where pharmacist staffing is limited and full review coverage is difficult to sustain; the collaborative approach markedly improves efficiency, substantially reduces missed detections, and offers a practical solution for medication safety oversight in such settings.

### Limitations

The 213-prescription sample reflected an administrative rule rather than an a priori power calculation; we report Wilson-score 95% CIs so that estimate precision is directly visible. Subgroup intervals are wide, and the therapeutic duplication (n=5, 2.3%) and dosage inappropriateness (n=11, 5.2%) findings should be treated as exploratory pending validation in larger multicenter samples covering uncommon combinations and special populations. Both reviewers were junior pharmacists (1-2 years’ experience), so the AI-related gain may be smaller for senior staff and should not be extrapolated across seniority without further study. The 2-period crossover with a 2-week washout balanced period and rater effects but cannot fully exclude asymmetrical learning: knowledge acquired under AI assistance may have raised later unaided performance, biasing the estimated benefit toward the null and rendering the reported improvement, if anything, conservative.

The system itself has intrinsic scope limits. It analyzes each prescription in isolation, with no access to longitudinal data such as prior medications, allergies, or renal and hepatic function; dosage and special-population review were therefore judged only against package-insert dose ranges, indications, and population statements alongside the recorded diagnosis, and cumulative polypharmacy was not evaluated. Some dosing decisions cannot be fully adjudicated without laboratory parameters. The 14-billion-parameter Qwen3 model is less capable than leading proprietary models, which may constrain deep-reasoning tasks; moreover, the model ran under 4 bits (Q4_K_M) quantization, which may modestly reduce reasoning performance or alter hallucination behavior relative to full-precision weights, so the stand-alone results reported here reflect a quantized deployment and may represent a performance floor. Even so, the collaborative gains achieved under local deployment indicate that scale is not the sole determinant of assistive value. Inference took 15 to 30 seconds per prescription on consumer-grade hardware. Because this inference was performed as a batch preprocessing step before the review session, the latency was not included in the recorded per-prescription review time and did not affect the efficiency comparison; it would, however, lengthen overall batch turnaround and could become a bottleneck at very high outpatient volumes, where more capable hardware, batched processing, or parallel inference would be required. Beyond hardware, local deployment carries recurring operating costs that we did not quantify, including server maintenance, electricity, and the staff time needed to keep the package-insert knowledge base current. We also did not perform a formal economic evaluation, which should precede any claim about affordability or cost-effectiveness. The 583-entry knowledge base is a subset of the hospital’s formulary; off-base drugs receive no knowledge injection and are routed for manual review, so generalizability depends on continued expansion both locally and at sites with broader formularies. The reference standard classified 29.1% (62/213) of sampled prescriptions as inappropriate, a rate that reflects the 0.1% administrative sampling of a tertiary teaching hospital and may exceed the prevalence in many outpatient or primary care settings. Because positive predictive value falls as prevalence declines, the same model operating at a lower error prevalence would return a larger proportion of false-positive alerts: for the stand-alone system (sensitivity approximately 100%, specificity approximately 90%), illustrative positive predictive value decreases from approximately 80% at the observed prevalence to approximately 53% at a prevalence of 10% and 35% at 5%. A higher false-alarm fraction could aggravate alert fatigue and automation complacency, although the model’s high specificity limits the absolute number of false alerts and the human-in-the-loop design allows pharmacists to filter them; characterizing the real-world alert burden will therefore require prospective evaluation across settings with differing baseline error rates.

Measurement issues also apply. The absolute hallucination counts were small (42 plain and 10 augmented), limiting the precision of the type distribution; severity was not graded, and a clinical-significance taxonomy would enable finer comparison of augmentation strategies. Only explicit problem statements were adjudicated, so hallucinations embedded in justifications for prescriptions deemed appropriate were missed, making the per-prescription rate probably conservative. The sample contained no confirmed drug-drug interaction or physicochemical incompatibility error, leaving proficiency in these clinically critical domains unvalidated despite prompt coverage. The post hoc sensitivity analysis was unblinded, introducing some incorporation bias, and is reported only as a robustness check.

Finally, the collaborative efficiency advantage partly reflected preflagging of suspect prescriptions, raising a real risk that busy pharmacists rely on alerts and underscrutinize unflagged orders. Safe deployment therefore requires AI alerts to supplement rather than replace complete pharmacist review, with workflow safeguards, periodic audit of nonflagged prescriptions, and targeted training to manage automation complacency before broader implementation.

### Future Directions

Future work should pursue several complementary directions. Domain-specific fine-tuning informed by expert-reviewed prescriptions and pharmacist feedback could further refine accuracy for particular drug classes [16,17]. Deeper integration with the hospital information system would enable automated retrieval of laboratory results, allergy history, and medication history, supporting genuinely patient-level rather than prescription-level analysis. A closed-loop learning mechanism that incorporates pharmacist accept or reject decisions could progressively improve advisory quality. Privacy-preserving, deidentified sharing of knowledge bases and exemplar cases across institutions also warrants exploration. If licensing and offline deployment allow, adding richer resources such as Micromedex or UpToDate to the package-insert knowledge base could broaden indication and dosage coverage.

### Conclusions

In this study, the pharmacist was kept at the center of decision-making, and a prescription-review decision-support system was built on the Qwen3-14B LLM, deployed inside the hospital through the Ollama platform together with a structured drug knowledge base. The crossover evaluation showed that AI assistance increased pharmacist review accuracy from 82.6% to 97.2% and sensitivity from 55% to 98% (both *P*<.001), while shortening the per-prescription review time from 2.33 (SD 0.97) to 1.12 (SD 0.49) minutes, an approximately 51.9% reduction (Wilcoxon signed-rank *Z*=−12.65; *P*<.001; *r*=0.87; N=213). Knowledge augmentation reduced the model hallucination rate from 19.7% to 4.7% (relative reduction 76.2%). By keeping all prescription data within the hospital’s own network, local deployment offers health care institutions a workable and secure way to integrate AI-driven pharmacy services without stepping outside their existing data-compliance requirements.

## Data Availability

The datasets analyzed in this study are deidentified routine clinical records held by the institution and cannot be released openly under the hospital’s data-governance policy. The deidentified dataset may be shared for noncommercial academic use upon reasonable request to the corresponding author, subject to a data-use agreement and relevant institutional approvals.

## Appendix

Multimedia Appendix 1 Hardware and software specifications, additional details of the reference standard, and additional statistical analysis details.

Multimedia Appendix 2 Verbatim prompt template used by the locally deployed large language model for outpatient prescription review (with the Chinese original and English translation), including the role definition, task specification, knowledge context, and output format components.

## Abbreviations

LLM: large language model
RAG: retrieval-augmented generation
