# Supplementary material for: Locally Deployed Large Language Models for AI-Assisted Outpatient Prescription Review: Crossover Study
Source: JMIR Med Inform. 2026 Sep 14;14:e97520. doi: 10.2196/97520 (PMC13620432; doi:10.2196/97520)
Supplement: Supplementary file 1 [file medinform_v14i1e97520_app1.docx]

# Multimedia Appendix 1

**Table A1.** Prescription-review dimensions specified in the task-specification component of the prompt template.

| **Review dimension** | **What is assessed** |
| --- | --- |
| Indication–diagnosis correlation | Whether the drug’s labeled indication matches the recorded diagnosis |
| Dosage and administration | Whether the dose and frequency fall within the insert’s recommended range;Whether the administration route is consistent with the insert |
| Contraindications | Whether the patient has any contraindication to the prescribed drug listed in the insert |
| Treatment duration | Whether the length of the treatment course is appropriate |
| Drug–drug interactions | Whether co-prescribed drugs carry documented interactions |
| Incompatibility | Physicochemical incompatibility among combined drugs or with the solvent |
| Therapeutic duplication | Duplicate agents or overlapping drugs of the same therapeutic class |
| Solvent choice | Appropriateness of the selected diluent or solvent |
| Special populations | Insert precautions for special populations (e.g., pregnancy, lactation, pediatric, geriatric) |

**Implementation details.**

The server ran Windows 10 with an Intel i7-8700 CPU, 32 GB RAM, and an NVIDIA RTX 3060 GPU (12 GB VRAM). The Qwen3-14B model used Q4_K_M quantization; greedy decoding (temperature = 0) generated up to 5120 tokens per prescription.

**Reference standard: additional details**

In our institution, more than five years of review experience together with the rank of supervising pharmacist is the minimum requirement for performing prescription review independently and for adjudicating disputed cases; this threshold was therefore considered adequate for constructing a working reference standard, while we recognize that an expert consensus is itself an imperfect proxy for absolute ground truth (addressed in the Limitations). Expert judgments were performed concurrently with the experimental judgments; the experts were not allowed to see any results of the pharmacist reviews or the AI system output until after their own judgments, which assured complete independence between the reference standard and the approaches being evaluated.

Hallucination adjudication and the prescription-appropriateness reference standard were two independent assessment channels. Hallucination evaluation checked the correspondence between AI outputs and the package inserts or recommendations, not prescription appropriateness per se. The experts evaluated AI outputs and adjudicated hallucinations only after the reference standard had been completed, without compromising its independence.

The independently developed reference standard removes incorporation bias from the primary analysis, yet it may still classify as appropriate a few inappropriate prescriptions that the experts did not detect. Because such misclassification is present equally across all conditions, the direction of conclusions is preserved. For the sensitivity analysis, any prescription flagged by one or more conditions but classified as appropriate under the reference standard was returned to the expert panel for post hoc review. During this review blinding was lifted and experts had access to the original flags; we acknowledge this constitutes partial incorporation of index-test results and therefore report the analysis only as a robustness check. Verified inappropriate prescriptions were added to an extended reference standard, and all performance measures were re-analyzed.

## Statistical analysis: additional details

The primary outcome was overall review accuracy (the number of correctly classified prescriptions divided by the total number of prescriptions). Secondary outcomes were sensitivity (the proportion of inappropriate prescriptions correctly detected), specificity (the proportion of appropriate prescriptions correctly identified), the false-positive rate, the false-negative rate, and the mean review time per prescription. Between-condition comparisons used the McNemar test with Yates continuity correction; subgroup comparisons, where cell counts were small, used the exact (binomial) McNemar test. The same paired tests were applied to the plain-versus-augmented AI hallucination comparison, and to the comparison of standalone knowledge-augmented AI with the unaided condition. All proportions are reported as point estimates with Wilson-score 95% confidence intervals (CIs). Cohen’s kappa served two distinct purposes, both interpreted against the Landis and Koch criteria. Applied to the unaided pharmacist judgments, it compared each pharmacist’s classifications with the reference standard; since P1 and P2 reviewed different, non-overlapping batches, the resulting values quantify agreement with the reference standard (criterion agreement) rather than inter-rater reliability between the two pharmacists. Applied to the hallucination adjudication, where the two senior pharmacists independently rated the same model outputs, kappa did measure inter-rater reliability in the usual sense.

Before the two periods were pooled, we tested for a sequence (carryover/period) effect by comparing, between the two sequences, the per-prescription total of the outcomes recorded across the two periods, using the Mann-Whitney U test; *P* > .10 was treated as evidence against a meaningful effect. Because the paired unit in this design is the prescription rather than the pharmacist, and the two sequences correspond to the two prescription batches, this comparison primarily verifies that the batches were exchangeable and that no sequence-level effect was present rather than isolating a strict person-level carryover effect; the latter was addressed by the two-week washout and is considered further in the Discussion. Note also that, because the two conditions for each prescription were performed by different reviewers, the prescription-level paired differences carry a rater component; the counterbalanced crossover design balances this component across conditions in the aggregate. As each prescription was reviewed under both conditions, the data are paired; accuracy, sensitivity and specificity were compared between conditions using the paired McNemar test (with continuity correction), and the between-condition accuracy difference is reported with its 95% CI.

The only pre-specified primary endpoint was the between-condition difference in overall accuracy (pharmacist-alone vs human-AI collaboration); all other endpoints, including AI-modality and subgroup comparisons, were exploratory, and no adjustment for multiplicity of comparisons was made. For each McNemar comparison the discordant-pair counts are reported so that the test statistic can be verified. The sensitivity analysis incorporated the additional expert-confirmed inappropriate prescriptions into an extended reference standard and repeated all analyses. Every test was two-tailed at the .05 significance level. Because the sample size was fixed by the administrative sampling rule, no power calculation was used to justify it, and the fixed sample size is acknowledged as a limitation.

Review times were summarized as mean with SD and median with IQR. For each prescription, the within-prescription difference (pharmacist alone minus collaborative) was calculated, with its mean, 95% CI, and percentage reduction reported. Because the paired differences departed from normality (Shapiro-Wilk test), the Wilcoxon signed-rank test served as the primary comparison, with a confirmatory paired t-test. Effect sizes were reported as r for the Wilcoxon test and Cohen’s dz for the t-test. All tests were two-sided, with P less than .05 considered significant.
